# Successor to Mr. Liston

**Published:** 1848-02

**Authors:** 


### SUCCESSOR TO MR. LISTON.

Professor Syme, of Edinburgh, has been appointed Professor of Surgery at University College Hospital, London. The appointment is well spoken of in the journals. None other than an able man should presume to occupy the place of Robert Liston.

